# An Atypical Manifestation of Necrotizing Fasciitis in a Patient With Type II Diabetes

**DOI:** 10.7759/cureus.54062

**Published:** 2024-02-12

**Authors:** Dawnica Nadora, Denise Nadora, Daniel I Razick, Eldo Frezza

**Affiliations:** 1 Dermatology, California Northstate University College of Medicine, Elk Grove, USA; 2 Internal Medicine, California Northstate University College of Medicine, Elk Grove, USA; 3 Surgery, California Northstate University College of Medicine, Elk Grove, USA

**Keywords:** emergent fasciotomy, spider bite, diabetic foot infection, types 2 diabetes, necrotizing fascitis

## Abstract

In this case report, we discuss a 32-year-old diabetic male patient who presented with right foot pain three days following a spider bite. The foot progressively became swollen, preventing the patient from bearing weight on it. After admission to the emergency department, the examination showed discoloration of the dorsum of the proximal phalanx of the first toe with an open wound and pus. The patient received fluid resuscitation along with a course of metronidazole and levofloxacin. Subsequently, the patient was referred to an orthopedic and podiatric team where he underwent a complete foot fasciotomy. The procedure was successful, and the patient recovered well. This case showcases a rare manifestation of necrotizing fasciitis (NF) and highlights the importance of future research regarding NF and its association with diabetes mellitus.

## Introduction

Necrotizing fasciitis (NF), also known as flesh-eating disease, is a rare and aggressive infection of the deep fascia. It is commonly a secondary condition, caused by bacterial infection following an injury that produces an opening in the epidermis of the skin [1]. The disease is diagnosed when the bacteria has spread throughout the adjacent tissues of the deep fascia, leading to ischemia and gangrene of the surrounding tissues, including the subcutaneous fat and dermis. Common causal bacteria include gas-producing organisms such as Clostridium and toxin-producing bacteria such as Staphylococcus aureus and Streptococcus pyogenes. These groups of bacteria can present as gas gangrene and sepsis, respectively [2].

The patient discussed in this case report has type II diabetes mellitus (T2DM), which is caused by increased insulin resistance and followed by a decrease in insulin secretion by pancreatic beta cells [3]. T2DM presents with a significant complication of atherosclerosis of the small blood vessels in the legs and feet [4]. The leading risk factor for NF is diabetes mellitus (DM) owing to its various associated symptoms that can contribute to the pathology of NF, including peripheral neuropathy, foot ulcers, and increased susceptibility to infections [4]. We present a rare case of a patient with T2DM who suffered from NF secondary to a spider bite.

## Case presentation

A 32-year-old male with a five-year history of T2DM (controlled with metformin) presented to the emergency department (ED) with progressively worsening pain in his right foot. He reported to have been bitten by a common spider, specifically not a brown recluse, and subsequently experienced swelling in his foot. Because of his diabetic neuropathy, the patient had poor sensation in his foot and did not feel the spider bite. The patient also reported no sensation or tenderness of the foot. Vital signs are summarized in Table 1, and notable laboratory findings upon arrival are summarized in Table 2. 

**Table 1:** Vital signs upon arrival

	Value	Reference Range
Temperature (Degrees Fahrenheit)	101.2	97-99
Blood Pressure	90/60	120/80
Heart Rate	118	60-100
Height (Inches)	69	Variable
Weight (Kilograms)	113.4	Variable

**Table 2:** Laboratory values upon arrival

	Value	Reference Range
White Blood Cell count (WBCs/µL)	20,200	4,500-11,000
Blood Glucose (mg/dL)	410	Variable (70-100 when fasted)
Lactate (mmol/L)	5	<2

**Figure 1:**
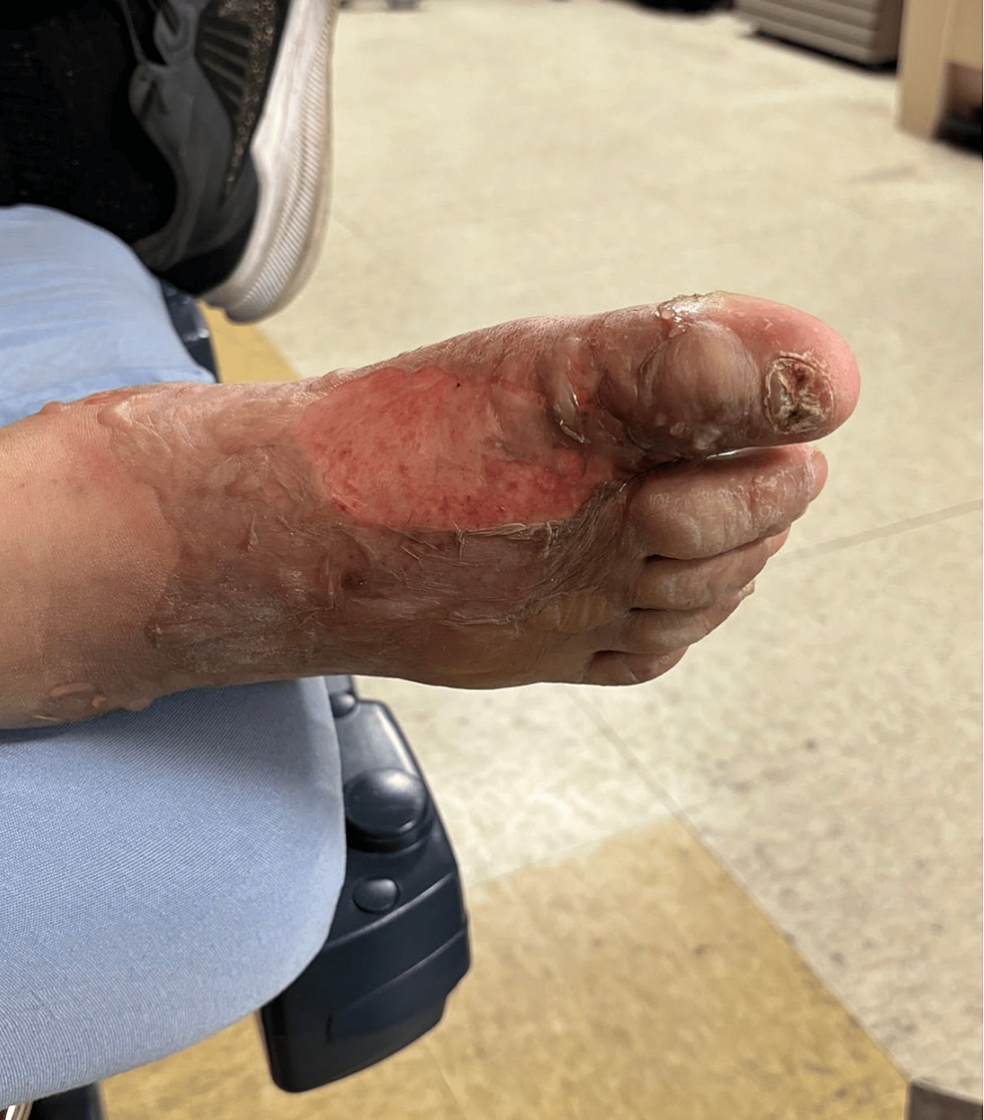
Medial dorsal aspect of the right foot with nearly complete exfoliation

The physical examination showed a swollen foot with an open wound and pus on the dorsal side of the proximal phalanx of the first toe. Crepitus was detected upon palpation of the first toe. The medial portion of the dorsum of the foot showed complete exfoliation of the skin (Figure 1).

**Figure 2:**
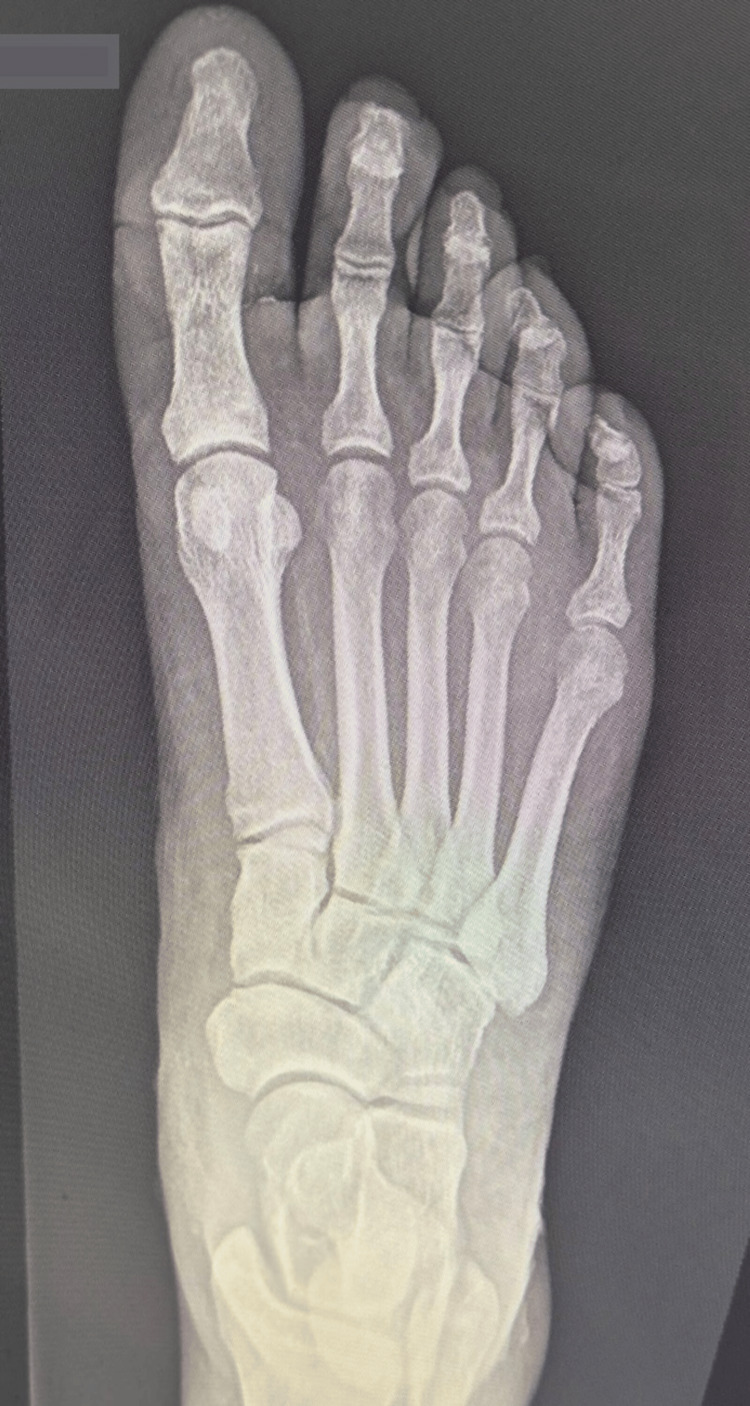
Dorsoplantar right foot X-ray with no signs of air pockets

The plantar side of the foot appeared to be swollen; however, there was no exfoliation or crepitus upon examination. A dorsoplantar X-ray did not show the presence of air in the foot (Figure 2).

**Figure 3:**
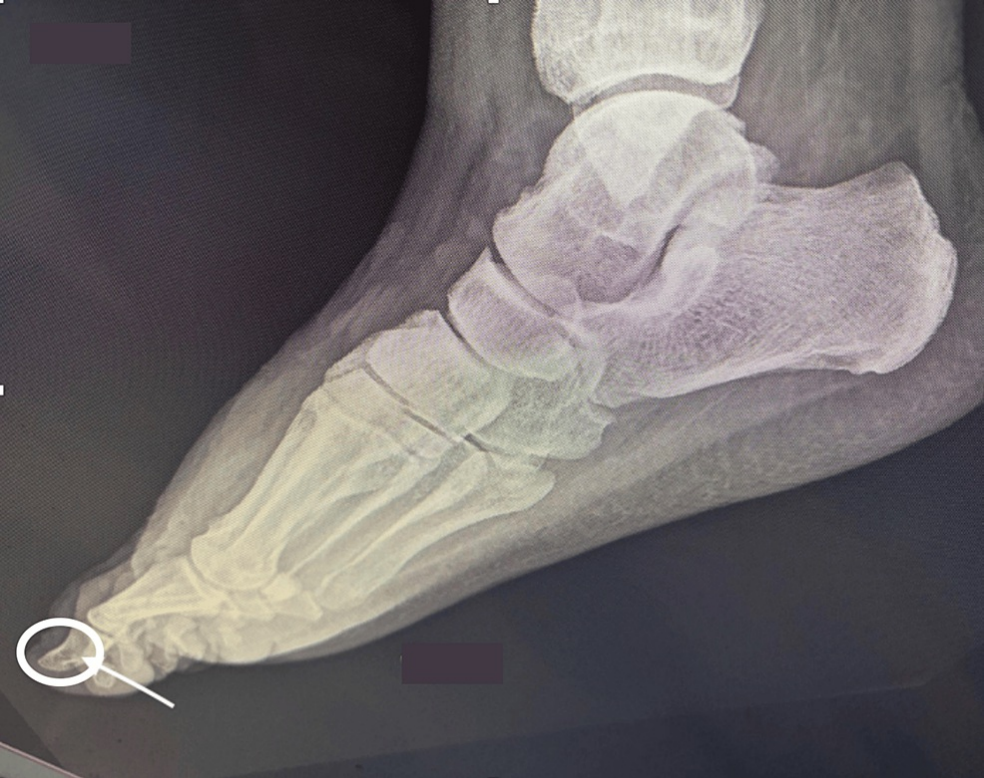
Lateral view of the right foot X-ray with a subtle air pocket in the distal aspect of the first phalanx Air pocket encircled. The distal aspect of the first phalanx is indicated by an arrow.

However, a lateral view indicated a subtle air pocket at the level of the first phalanx (Figure 3).

Given the patient was in septic shock, he underwent fluid resuscitation and was given a two-gram dose of metronidazole and prescribed a ten-day course of 500 milligrams of levofloxacin to be taken once daily. Following this, he was referred to a collaborative orthopedic and podiatry team, where a complete foot fasciotomy was performed. The patient recovered well after the surgery. 

## Discussion

NF, although rare, is a severe soft-tissue infection because of rapidly spreading inflammation affecting subcutaneous soft tissue with skin gangrene and vascular thromboses [1]. Infection is usually caused by trauma, but it could also stem from a simple scrape or an insect bite, as seen in the present case study. The inflammation and necrosis are typically incited by a toxin-producing, virulent bacteria, classically from group A streptococcus, affecting primarily immunocompromised patients such as those with DM, morbid obesity, alcoholism, advanced atherosclerotic disease, and decubitus ulcers [2,5]. Moreover, NF is characterized by severe systemic toxicity as also observed with the present patient because of his febrile condition upon admission to the ED. NF can easily be misdiagnosed as a benign soft tissue infection; thus, prompt diagnosis is crucial as proper timing of surgical debridement is essential for a patient's survival.

Common risk factors identified for NF are DM, immunosuppressive therapy, chronic infectious diseases, and advanced age [1]. As indicated in the laboratory findings, the patient’s elevated glucose level of 410 mg/dL, in conjunction with their medical history of DM, predisposed the patient to NF. DM is a chronic medical condition that adversely affects the immune system because of long-term uncontrolled blood sugar, placing diabetic patients at a heightened risk for infection. Because of angiopathy and neuropathy caused by DM, the patient exhibited diminished sensation and was not aware of the spider bite.

This is an unusual case of NF as the superficial skin exfoliation observed in this patient presents similarly to lesions seen in acute cellulitis. The typical presentation for acute cellulitis includes a warm, erythematous, poorly demarcated area with associated edema and tenderness to palpation [6]. Moreover, inflamed tissue commonly involves blood supply loss and cellular dysfunction, leading to both cell and tissue death, as observed in both cellulitis and NF. As a result, hair follicles are generally unable to thrive in necrotic areas. Additionally, the imaging results appeared to be insignificant and failed to demonstrate any evidence of soft tissue gas, as shown in Figure 2. However, there is a slight air pocket visible at the level of the first phalanx from the lateral view, as depicted in Figure 3. Although the presence of soft tissue gas is commonly seen in NF patients, it is nonspecific to NF and could be observed in patients with conditions like gas gangrene, penetrating trauma, pneumomediastinum, and esophageal perforation [7,8].

First-line treatment for NF includes aggressive surgical debridement and broad-spectrum intravenous antibiotic therapy [5]. In certain cases, limb amputation may be considered if deemed necessary. Metronidazole and levofloxacin were administered as the patient was febrile and had an elevated WBC count. Furthermore, orthopedic surgeons are often involved as inflamed tissue in the lower extremities must be surgically removed [5]. As NF is notoriously tough to diagnose, timely diagnosis is critical. Immediate and intensive surgical debridement of affected tissue is key to the successful treatment of NF.

This case report has its limitations. Because of the unusual nature of this case presentation, generalizing the findings of the report given its uncommon occurrence and noteworthy features is difficult. Thus, this case serves to emphasize the importance of a careful analysis of a patient’s medical history, physical examination, and imaging to ensure accurate diagnoses, particularly with atypical presentations.

## Conclusions

This case serves as a warning regarding the simple underlying causes of NF in diabetic patients, such as a spider bite in this case report, as delayed treatment can lead to fatal outcomes. Moreover, any injury to the foot, may it be from simply stepping on a nail or cuts obtained during a manicure, must be promptly assessed to prevent necrotic tissue from developing, particularly in patients with DM. The patient's condition was effectively addressed through a comprehensive review of the medical history and appropriate administration of antibiotics and surgical intervention. Furthermore, this case underscores the importance of evaluating any foot injury with proper diagnosis to avoid NF and differentiating NF from other soft tissue infections that might obscure the clinical picture. Numerous factors can influence the clinical diagnostic accuracy of NF, emphasizing the critical role of a thorough history, physical examination, and the contribution of imaging results in assessing atypical presentations.
